# Alterations of Excitation–Contraction Coupling and Excitation Coupled Ca^2+^ Entry in Human Myotubes Carrying *CAV3* Mutations Linked to Rippling Muscle Disease

**DOI:** 10.1002/humu.21431

**Published:** 2011-02-03

**Authors:** Nina D Ullrich, Dirk Fischer, Cornelia Kornblum, Maggie C Walter, Ernst Niggli, Francesco Zorzato, Susan Treves

**Affiliations:** 1 Department of Physiology, University of BernBern, Switzerland; 2 Department of Neuropediatrics, University Children's Hospital BaselSwitzerland; 3 Department of Neurology Basel University HospitalBasel, Switzerland; 4 Department of Neurology, University of BonnBonn, Germany; 5 Friedrich-Baur-Institute, Department of Neurology, Ludwig-Maximilians UniversityMunich, Germany; 6 Departments of Anesthesia and Biomedizin, Basel University HospitalBasel, Switzerland; 7 Dipartimento di Medicina Sperimentale e Diagnostica, sez Patologia Generale, University of FerraraFerrara, Italy

**Keywords:** caveolin-3, *CAV3*, rippling muscle disease, excitation–contraction coupling, excitation coupled Ca^2+^ entry, Ca^2+^ homeostasis, TIRF microscopy

## Abstract

Rippling muscle disease is caused by mutations in the gene encoding caveolin-3 (CAV3), the muscle-specific isoform of the scaffolding protein caveolin, a protein involved in the formation of caveolae. In healthy muscle, caveolin-3 is responsible for the formation of caveolae, which are highly organized sarcolemmal clusters influencing early muscle differentiation, signalling and Ca^2+^ homeostasis. In the present study we examined Ca^2+^ homeostasis and excitation–contraction (E-C) coupling in cultured myotubes derived from two patients with Rippling muscle disease with severe reduction in caveolin-3 expression; one patient harboured the heterozygous c.84C>A mutation while the other patient harbored a homozygous splice-site mutation (c.102+ 2T>C) affecting the splice donor site of intron 1 of the *CAV3* gene. Our results show that cells from control and rippling muscle disease patients had similar resting [Ca^2+^]_i_ and 4-chloro-*m*-cresol-induced Ca^2+^ release but reduced KCl-induced Ca^2+^ influx. Detailed analysis of the voltage-dependence of Ca^2+^ transients revealed a significant shift of Ca^2+^ release activation to higher depolarization levels in *CAV3* mutated cells. High resolution immunofluorescence analysis by Total Internal Fluorescence microscopy supports the hypothesis that loss of caveolin-3 leads to microscopic disarrays in the colocalization of the voltage-sensing dihydropyridine receptor and the ryanodine receptor, thereby reducing the efficiency of excitation–contraction coupling. Hum Mutat 32:309–317, 2011. © 2011 Wiley-Liss, Inc.

## Introduction

Rippling muscle disease (RMD; MIM♯ 606072) is a rare autosomal dominant disorder caused by mutations in *CAV3* (MIM♯ 601253) the gene encoding caveolin-3 (CAV3), a caveolin isoform exclusively expressed in skeletal, cardiac, and smooth muscles [Betz et al., 2001; Woodman et al., 2004]. Caveolins are small 22-kDa transmembrane proteins that homo-oligomerize on the plasma membrane giving rise to caveolae, or invaginated structures of 50–100 nm in diameter (for recent reviews, see [Cohen et al., 2004; Hansen and Nichols, 2010; Hnasko and Lisanti, 2003]). In skeletal muscle numerous proteins including β-dystroglycan, nitric oxide synthase, phosphofructokinase, tubulin, cadherin-M converge within sarcolemmal caveolae [Galbiati et al., 2001; García-Cardena et al., 1997; Song et al., 1996; Sotgia et al., 2000, 2003; Volonte et al., 2003), whereas in mature muscle fibers, caveolins are also distributed in the subsarcolemmal space on the neck of the T-tubules, where ion channels, pumps, kinases, and signaling molecules collect [Kristensen et al., 2008; Lamb, 2005; Murphy et al., 2009; Scriven et al., 2005]. Besides functioning as a converging molecule, CAV3 is involved in myoblast differentiation, survival, and cell fusion, and its transcription level increases early in development during muscle tissue differentiation [Galbiati et al., 2001; Volonte et al., 2003]. Experiments on zebrafish have demonstrated that injection of embryos with CAV3 antisense morpholinos results in embryos with uncoordinated movements probably due to disorganized fused myoblasts, chaotic filament bundles of the contractile proteins, dispersed mitochondria and poorly developed T-tubules [Nixon et al., 2005].

Although their exact physiological role is not clear, the above data indicate that caveolin-3 plays an important role in muscle function and mutations in *CAV3* have indeed been linked to several hereditary myopathies, among which are Limb Girdle Muscular Dystrophy (LGMD; MIM♯ 607801), Rippling Muscle Disease (RMD; MIM♯ 606072), Distal myopathy (DM; MIM♯ 601253), and HyperCKemia [Betz et al., 2001; Gazzerro et al., 2010; Woodman et al., 2004]. In some cases, mutations in *CAV3* have also been associated with cardiomyopathy [Calaghan and White, 2006; Catteruccia et al., 2009; Hayashi et al., 2004; Vatta et al., 2006]. *CAV3* maps on human chromosome 3p25 and is made up of two exons; so far, 24 missense mutations, 1-bp insertion, 3-bp deletions, a splice-site substitution, and a genomic macro deletion have been reported in patients with caveolinopathies [Aboumousa et al., 2008; Woodman et al., 2004]. Most mutations are inherited in a dominant way and lead to a severe decrease in the expression of all CAV3, because mutated and wild-type proteins multimerize within the Golgi, where they form a complex that is tagged for proteolysis and degraded in the proteosome leading to very low levels of expression of caveolin-3 on the sarcolemma [Cohen et al., 2004; Galbiati et al., 1999]. CAV3 is made up of 151 amino acids, of which the first 55 residues constitute the NH_2_ terminus, residues 56–73 make up the scaffolding domain important in homo-oligomerization, residues 76–108 form the transmembrane domain that gives rise to a hair loop structure, allowing the COOH-and NH_2_-teminus to face the same side of the membrane [Cohen et al., 2004; Galbiati et al., 2001]. Mutations found in patients are more frequent in the NH_2_ domain, followed by the scaffolding and membrane domains [Aboumousa et al., 2008; Woodman et al., 2004]. Interestingly, clinical evidences have demonstrated that the same *CAV3* mutation in different populations and even within the same family, can result in a different clinical phenotype, indicating the influence of additional factor(s) in the phenotypic outcome of the mutation.

Recently, Fischer et al. [2003] identified a mutation in *CAV3* in a large German family. This family harbored the c.84C>A heterozygous substitution leading to the p.D28E mutation. Another German family was subsequently identified harboring an autosomal recessive splice site mutation c.102+ 2T>C in intron 1 [Müller et al., 2006]; both mutations lead to drastically reduced levels of expression of CAV3 in the skeletal muscle. The patient harboring the p.D28E mutation had clear signs of RMD characterized by percussion-induced rapid muscle contraction and muscle mounding, painful muscle cramping, elevated creatine kinase levels, and hypertrophic calves [Fischer et al., 2003], whereas the patient harboring the splice-site mutation had muscle weakness, elevated creatine kinase levels, percussion-induced muscle mounding, and mild myopathic degeneration with fiber-size variation and an increase of connective tissue [Müller et al., 2006].

Although the precise pathomechanism is still elusive, a number of reports have indicated that CAV3 may have a role in Ca^2+^ homeostasis [Calaghan and White, 2006; Kamishima et al., 2007; Weiss et al., 2008], and it has been shown that expression of mutated caveolin in muscle cells alters the function of the dihydropyridine receptor (DHPR) [Couchoux et al., 2007]. More recently, it was demonstrated that CAV3 interacts directly with the ryanodine receptor (RyR1) sarcoplasmic reticulum Ca^2+^ release channel [Vassilopoulos et al., 2010]. In the present report we studied excitation–contraction coupling in human skeletal muscle myotubes obtained from the two patients harboring the above-described *CAV3* mutations. Our results show that the myotubes from control or RMD patients have similar resting [Ca^2+^] and ryanodine receptor-activated Ca^2+^ release. Interestingly however, cells bearing the mutated *CAV3* showed a shift in depolarization-induced Ca^2+^ release and a decreased depolarization-induced Ca^2+^ influx, suggesting that lack of caveolin leads to a less efficient excitation–contraction coupling.

## Materials and Methods

### Mutation Nomenclature

Nucleotide numbering for *CAV3* reflects cDNA numbering with +1 corresponding to the A of the ATG translation initiation codon in the reference sequence (GenBank NM_033337.1), according to journal guidelines (http://www.hgvs.org/mutnomen). The initiation codon is codon 1.

### Primary Skeletal Muscle Cultures

Primary skeletal muscle cultures were established from fragments of muscle biopsies obtained from patients undergoing diagnostic testing, as previously described [Ducreux et al., 2004]. Cells were cultured on 0.17-mm thick glass coverslips in growth medium and induced to differentiate into myotubes by culturing them in DMEM plus 4.5 mg/ml glucose, 0.5% bovine serum albumin (BSA), 10 ng/ml EGF, 0.15 mg/ml creatine, 5 ng/ml insulin, 200 mM glutamine, 600 ng/ml penicillin G and streptomycin, and 7 mM HEPES, pH 7.4 for 7–10 days.

### Cytoplasmic Calcium Measurements

Coverslip grown myotubes were loaded with the fluorescent ratiometric Ca^2+^ indicator fura-2-AM (final concentration 5 µM) in differentiation medium for 30 min at 37°C, after which the coverslips were mounted onto a 37°C thermostatically controlled chamber that was continuously perfused with Krebs-Ringer medium. On-line measurements were recorded using a fluorescent Axiovert S100 TV inverted microscope (Carl Zeiss GmbH, Jena, Germany) equipped with a 20 × water-immersion FLUAR objective (0.17 NA), filters (BP 340/380, FT 425, BP 500/530), and attached to a Hamamatsu multiformat CCD camera. Images were acquired at 1-sec intervals and the exposure time was fixed at 100 msec for both (340-and 380-nm excitation) wavelengths. Changes in fluorescence were analyzed using an Openlab imaging system and the average pixel value for each cell was measured at excitation wavelengths of 340 and 380 nm as previously described [Ducreux et al., 2004]. Individual cells were stimulated by means of a 12- or 8-way 100 mm diameter quartz micromanifold computer controlled microperfuser (ALA Scientific instruments, Westbury, NY), as previously described [Ducreux et al., 2004].

### Ca^2+^ Influx by TIRF Microscopy

Depolarization-induced Ca^2+^ influx was monitored by TIRF microscopy (total internal fluorescent microscopy) in myotubes loaded with fluo-4-AM. Briefly, glass coverslips grown and differentiated human myotubes were mounted on a thermostated perfusion chamber, bathed continuously in Krebs-Ringer buffer. Excitation-coupled Ca^2+^ entry (ECCE) [Bannister et al., 2009; Cherednichenko et al., 2004] was measured after application of 60 mM KCl to myotubes pretreated with 100 µM ryanodine to block RyR1-mediated Ca^2+^ release. On-line fluorescence images were acquired using an inverted Nikon TE2000 TIRF microscope equipped with an oil immersion CFI Plan Apochromat 60 × TIRF objective (1.49 N.A.) and an electron multiplier Hamamatsu CCD camera C9100-13, which allows fast data acquisition, as previously described [Treves et al., 2010]. Our TIRF microscope is equipped with a surface reflective interference contrast (SRIC) cube to identify the focal plane corresponding to the coverglass/cell membrane contact prior to TIRF acquisition. The focus was maintained at the coverglass/cell membrane contact by using the perfect focus system (PFS) that exploits an infrared laser beam and a quadrant diode for the online control of the microscope's focusing motor. Fluo-4-loaded cells were excited with a solid-state laser beam at 488 nm and the emitted fluorescence was collected through a 520 narrow band filter. Data were analyzed using Metamorph imaging software (Molecular Devices, Menlo Park, CA).

### Electrophysiological Measurements and Confocal Ca^2+^ Imaging

Human myoblasts were grown on laminin-coated glass coverslips and differentiated into myotubes. Cells were voltage-clamped in the whole-cell patch clamp configuration with low resistance borosilicate glass micropipettes (1–3 MΩ) using an Axopatch 200B amplifier (Axon Instruments, Union City, CA) controlled by a custom-written data-acquisition software developed by LabView (National Instruments, Austin, TX). The pipette solution contained (in mM) 100 CsAsp, 20 tetraethylammonium(TEA)-Cl, 10 HEPES, 5 MgCl_2_, 5 Na_2_ATP, 0.05 EGTA, 0.1 K_5_-Fluo-3 at pH 7.2 (adjusted with CsOH). External solution contained (in mM) 130 CsCH_3_SO_3_, 2 MgCl_2_, 2 CaCl_2_, 10 Glucose, 20 HEPES at pH 7.4 (adjusted with CsOH). The voltage protocol consisted of stepwise depolarizations (50 msec) from a holding potential of − 80 mV to increasing potentials from − 60 mV to +10 mV. Activation of the voltage-dependent dihydropyridine receptor (skeletal DHPR Ca_V_1.1) triggered Ca^2+^ release from the SR via electromechanical coupling between DHPR and RyR1. Changes in [Ca^2+^]_i_ were simultaneously recorded with membrane currents using the fluorescent Ca^2+^ indicator K_5_-Fluo-3 (Biotium, Haward, CA) and a laser-scanning confocal microscope (MicroRadiance, BioRad, Hercules, CA) with a 60 × water immersion objective lens. Fluo-3 was excited at 488 nm with an argon ion laser, and emitted light was collected above 500 nm. Linescan images were recorded at a rate of 500 lines/sec. Confocal images were analyzed in ImageSXM (free software based on NIH Image) [Barrett, 2002] and further processed together with the voltage clamp data using IgorPro (Wavemetrics, Lake Oswego, OR). Changes in [Ca^2+^]_i_ are expressed as changes in fluorescence (Δ*F/F*_0_). All measurements have been performed at room temperature.

### Western Blotting

Total muscle homogenate and SR fraction obtained from skeletal muscle leftover fragments that had been stored in liquid N_2_ were isolated. Proteins were separated by SDS PAGE, blotted onto nitrocellulose, and probed with antibodies against caveolin-3 (GeneTex Inc., Irvine, CA; catalog No. GTX109650), RyR1 (Thermo Scientific, West Palm Beach, CA; catalog No. MA3-925), α1.1 subunit of the DHPR (Santa Cruz, Santa Cruz, CA; sc- 8160), SERCA2 (Santa Cruz, sc-8095), calsequestrin [Delbono et al., 2007], and glycogen phosphorylase (Santa Cruz, sc-4634), followed by peroxidase-conjugated secondary antibodies. Bands were visualized by chemiluminescence, using the Super Signal West Dura kit from Thermo Scientific. For comparison, the intensities of the immunoreactive bands were quantified by densitometry using Bio-Rad GelDoc 2000; intensities were corrected for glycogen phosphorylase (total muscle homogenate) and calsequestrin (total SR).

### Immunofluorescence Analysis

Glass coverslips grown and differentiated myotubes were fixed in an ice-cold solution of acetone:methanol (1:1) for 20 min, rinsed two times with phosphate-buffered saline (PBS), and blocked with 10% blocking buffer (Roche Applied Science, Indianapolis, IN) for 60 min at room temperature. Coverslips were incubated with goat anti-α1.1 subunit of the DHPR (final concentration 10 µg/ml) and mouse anti-RyR (final concentration 10 µg/ml) in PBS; after 60 min coverslips were rinsed three times 5 min each with PBS and incubated with donkey anti-goat FITC (Santa Cruz) for 60 min, extensively washed with PBS, and incubated with Alexa Fluor405 conjugated goat anti-mouse IgG (Invitrogen). After 60 min coverslips were washed and mounted in glycerol mounting medium. Fluorescence was visualized through a 100 × oil immersion CFI Plan Apochromat TIRF objective (1.49 NA), by exciting at 488 (Sapphire laser) to visualize FITC, and at 405 nm using a laser from Coherent Laboratories (Coherent Labs Inc., Santa Clara, CA). AlexaFluor 405 fluorescence was visualized using a BrightLine CH 427 filter (AHF Analysentechnir AG, Tubingen, Germany). Colocalization analysis of α 1.1 of the DHPR and RyR was performed using the colocalization application included in the Metamorph 5.7.4 software package as previously described [Treves et al., 2010]; only myotubes with more than two nuclei were analyzed.

### Statistical Analysis

Statistical analysis was performed using the Student's *t*-test for paired samples; means were considered statistically significant when the *P*-value was <0.05. The Origin computer program (Microcal Software, Inc., Northampton, MA) was used for statistical analysis.

## Results

**Figure 1:**
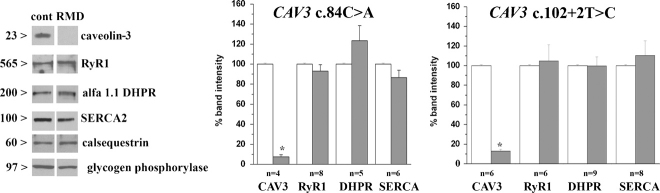
Western blot analysis of skeletal muscle proteins in muscle biopsies from the two RMD patients harboring *CAV3* mutations. Proteins (30 µg) in the total muscle homogenate (CAV3, α1.1 DHPR, RyR1 and glycogen phosphorylase) or total SR fraction (20 µg) (SERCA2 and calsequestrin) were blotted onto nitrocellulose and probed with the indicated antibodies as specified in the Methods section. The relative expression levels of the immunopositive bands in the biopsy from the RMD patient harboring the c.84C>A mutation (left) and the homozygous splice-site mutation c.102+ 2T>C (right) were compared to that of control biopsies that were considered 100%; intensity values were estimated by densitometric analysis of the indicated number of blots and normalized with respect to the band intensity of glycogen phosphorylase (total homogenate) or calsequestrin (SR). Bars represent mean ± SEM of n experiments; ^*^P<0.0001.

The aim of the present report is to assess whether mutations in *CAV3* lead to dysregulation of Ca^2+^ homeostasis in human muscle cells. In order to assess this we first monitored whether the absence/reduced levels of CAV3 affects the expression levels of the main components of the excitation–contraction (E-C) coupling machinery, namely, the DHPR and the RyR calcium channels, calsequestrin and the SERCA Ca^2+^ pump [Treves et al., 2009]. Figure 1A shows Western blot analysis of total muscle homogenate (CAV3, RyR1, DHPR) and of the total sarcoplasmic reticulum (SR) fraction (SERCA2, calsequestrin) obtained from muscle biopsy fragments of the patients harboring *CAV3* mutations and controls. As shown previously [Fischer et al., 2003; Müller et al., 2006], the amount of CAV3 present in the muscle homogenate of both patients is greatly reduced. Interestingly, no significant differences were found in the amounts of RyR1, DHPR α1.1, SERCA2, and calsequestrin expressed in the muscle biopsy from these patient compared to that expressed in control biopsies. Thus, the absence of caveolin-3 does not grossly alter the expression level of the protein components involved in Ca^2+^ homeostasis.

**Figure 2:**
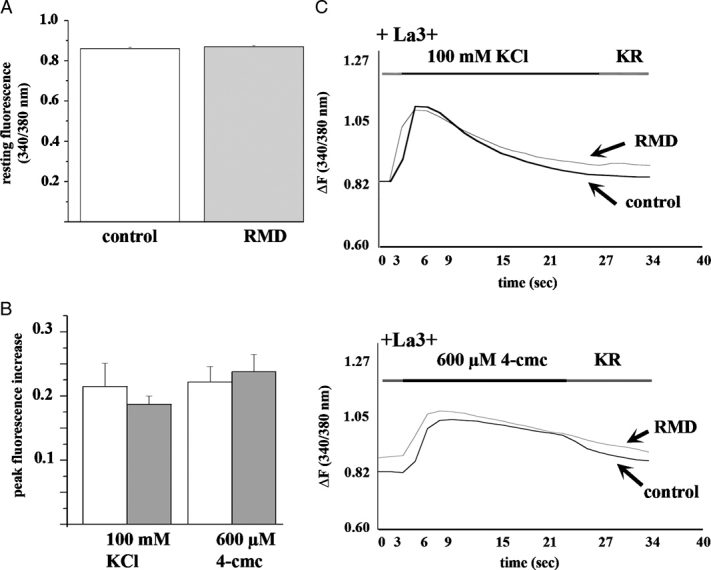
Characterization of cytoplasmic Ca^2+^ homeostasis in myotubes with CAV3 deficiency. Calcium imaging was performed in fura-2 loaded myotubes as described in the Methods section. **A:** Mean (± SEM of *n* = 58 and 92 for control and RMD, respectively) resting [Ca^2+^] (expressed as fluorescence intensity ratio 340/380 nm) was not different in control and RMD myotubes. **B:** Mean (± SEM) peak Ca^2+^ increase induced by the addition of 100 mM KCl (inducing depolarization) and 600 µM 4-chloro-*m*-cresol (4-cmc, RyR1 agonist) in the presence of Krebs-Ringer medium (KR) containing 100 µM La^3+^. Open boxes, control myotubes; gray boxes, myotubes from CAV3 deficient myotubes (*n* = 7–15 measurements). **C:** Traces showing fura-2 change in fluorescence (ratio 340/380 nm) of individual myotubes from a control and a *CAV3* mutation-bearing patient in response to 100 mM KCl and 600 µM 4-cmc.

We next studied the Ca^2+^ homeostasis of the myotubes from the two patients with RMD. Although myotubes were obtained from the two patients with different mutations, as shown in Figure 1A and reported for other *CAV3* mutations [Aboumousa et al., 2008; Woodman et al., 2004], both the c.84C>A and c.102+2T>C substitutions resulted in a drastic reduction of CAV3 expression. Because of this and because Western blot analysis revealed similar levels of expression of the main protein components involved in E-C coupling (Fig. 1), we pooled the results obtained on Ca^2+^ homeostasis on the myotubes from the two patients. Figure 2 shows that the mean resting fluorescent ratio and the peak Ca^2+^ release obtained by stimulating the cells with maximal amounts of either KCl (mimicking electrical depolarization) or 4-chloro-*m*-cresol (which directly activates the RyR1) [Zorzato et al., 1993] were not significantly different between control myotubes and myotubes from the two patients with RMD when the experiments were performed in Krebs Ringer +100 µM La^3+^, a general Ca^2+^ channel blocker used to prevent any contaminating Ca^2+^ influx. Panel C shows representative traces of Ca^2+^ release experiments performed in the presence of contaminating Ca^2+^ plus 100 µM La^3+^. These results indicate that the lack of CAV3 does not grossly affect RyR1-mediated Ca^2+^ release from the intracellular stores.

**Figure 3:**
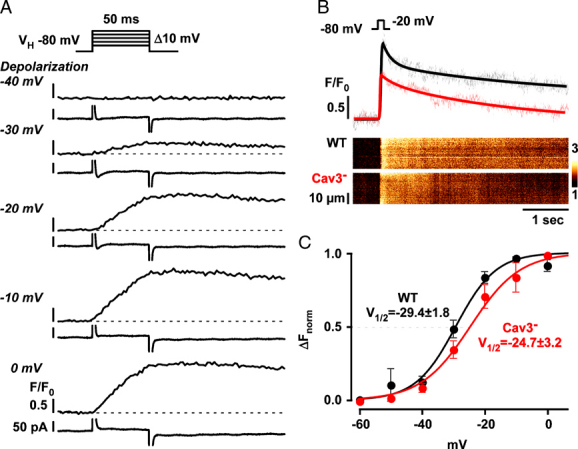
Voltage-dependence of Ca^2+^ transients in control and RMD myotubes. Cells were patch-clamped and held at a holding potential (*V*_H_) of − 80 mV. **A:** Paired sample traces of current (lower trace) and Ca^2+^ release recordings (upper trace) at different test potentials (from − 40 to 0 mV) in a fluo-3 loaded control myotube. Depolarizations (50 msec) to increasing membrane potentials activated Ca^2+^ release from the SR.**B:** Comparison of Ca^2+^ release during a depolarizing step to − 20 mV in control (black) and Cav-3 deficient RMD (red) myotubes. Linescan images and line profiles show the reduced Ca^2+^ transient amplitude in caveolin-3 deficient RMD myotubes at same trigger voltage when compared with control. **C:** Summary of the voltage-dependence of Ca^2+^ release in control (WT, *n* = 10) and caveolin-3-deficient RMD myotubes (*n* = 8). Ca^2+^ transient amplitudes have been normalized to the maximal release amplitude in each cell. Membrane potentials at half-maximal activation (V_1/2_) indicate a right shift of the voltage-dependence in RMD myotubes (*P*<0.05).

A more detailed investigation of the electromechanical coupling in control and RMD myotubes, however, revealed significant differences in the voltage-dependent Ca^2+^ release properties of cells from RMD patients. Using an electrophysiological approach combined with confocal Ca^2+^ imaging we studied Ca^2+^ release induced by membrane depolarization. Figure 3A shows original paired sample traces for current (lower trace) and Ca^2+^ transient (upper trace) in control human myotubes at increasing levels of membrane depolarization (for representative current traces see Supp. Fig. S1). As expected, increasing depolarization results in an increase in Ca^2+^ release, which saturates as the depolarizing voltage pulse reaches − 10 mV. Figure 3B shows the line profiles and corresponding linescan images of the Ca^2+^ response to a depolarizing step from − 80 to − 20 mV in control (upper black trace) and RMD myotubes (lower red trace), respectively. At the same trigger potential, RMD myotubes present lower voltage-induced Ca^2+^ release amplitudes from the SR when compared with control cells. The voltage-dependence of Ca^2+^ release is summarized in Figure 3C; normalized peak Ca^2+^ release amplitudes are plotted as a function of the test potential. The membrane potentials at half maximal Ca^2+^ release (V_1/2_) are indicated revealing a significant shift (*P*<0.05) in V_1/2_ from − 29.4 ± 1.8 mV in control myotubes to − 24.7 ± 3.2 in RMD myotubes. This right shift in V_1/2_ is further accompanied by a shift in the maximal Ca^2+^ transient amplitude from − 10 mV in control to 0 mV in RMD myotubes. These results indicate that depletion of CAV3 reduces the coupling efficiency between the DHPR and the RyR.

**Figure 4:**
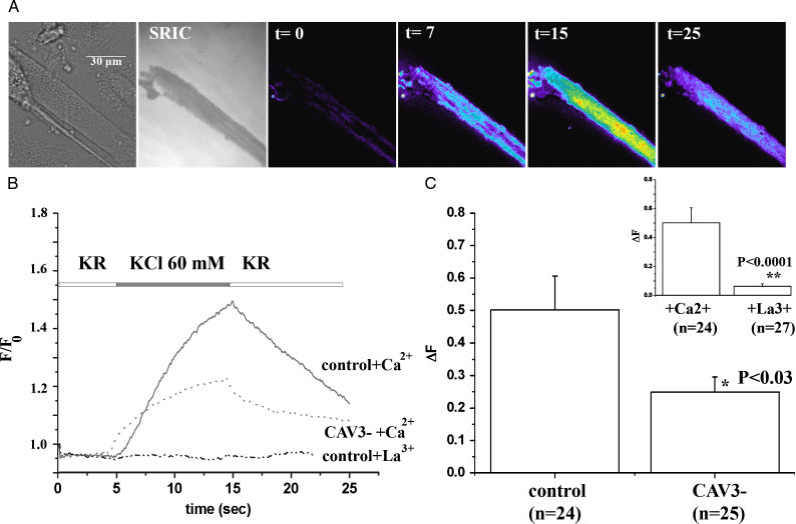
TIRF measurements of Ca^2+^ influx induced by 60 mM KCl in human myotubes. **A:** Myotubes from a control patient were visualized by brightfield (top left panel), with a surface reflection interference contrast (SRIC) filter to visualize and fix the focal plane of the coverglass/cell membrane interphase (top central panel). Next panels show pseudocolored ratiometric images (peak fluorescence after addition of KCl/ resting fluorescence) of fluo-4 fluorescence changes at the indicated time-points after application of KCl. Fluorescence was monitored through a 60 × TIRF objective and analyzed using Metamorph as detailed in the Methods section. Bar indicates 30 µm**. B:** Representative traces from ECCE showing changes in fluo-4 fluorescence in a myotube from a control individual (_____) and a patient harbouring a *CAV3* mutation (…….) stimulated with 60 mM KCl in the presence of 2 mM Ca^2+^ or myotubes from a control in the absence of added Ca^2+^ and in the presence of 100 µM La^3+^ (.-.-.-.-.). TIRF measurements were performed as indicated in the Methods section in myotubes pretreated with 100 µM ryanodine. **C:** Bar graph depicting mean (± SEM) peak increase of fluo-4 fluorescence induced by 60 mM KCl in control and caveolin-3 deficient myotubes in the presence of 2 mM Ca^2+^. Insert shows the mean (± SEM) peak fluo-4 fluorescence increase of control myotubes in the presence of 100 µM La^3+^ (gray bar) or 2 mM Ca^2+^ (empty bar).

Recently it was shown that in skeletal muscle myotubes, plasma membrane depolarization is accompanied by Ca^2+^ influx, which is mediated by the DHPR and has been defined as excitation coupled Ca^2+^ entry (ECCE) [Bannister et al., 2009; Cherednichenko et al., 2004]. Although the functional significance of this Ca^2+^ influx is currently unknown, it depends on the presence of both the RyR1 and DHPR. Becausev (1) depolarization-induced Ca^2+^ release is affected by the loss CAV3 (Fig. 3) [Couchoux et al., 2007], (2) studies by Vassilopoulos et al. [2010] demonstrated a direct interaction between CAV3 and the RyR1, and (3) ECCE depends on the presence of the DHPR and of the RyR, we studied ECCE in the myotubes from the two RMD patients. Figure 4 summarizes the pooled results obtained by TIRF microscopy on Ca^2+^ influx activated by 60 mM KCl. As indicated in the Methods section, myotubes were pretreated with 100 µM ryanodine to block Ca^2+^ release from the SR via RyR1 [Meissner, 1986]. The bottom trace in Figure 4B (.-.-.-) and the inset in Figure 4C show that in the absence of extracellular Ca^2+^ (and in the presence of 100 µM La^3+^) the addition of 60 mM KCl does not lead to a change in fluo-4 fluorescence, confirming that the increase in Fluo-4 is not due to calcium release from the SR. When the experiments were conducted in the presence of 2 mM Ca^2+^, on the other hand, the addition of KCl was accompanied by a transient increase in Fluo-4 fluorescence, confirming that this fluorescence increase represents Ca^2+^ influx from the extracellular medium. We then compared the extent of the KCl-activated Ca^2+^ influx in myotubes from the two RMD patients to that observed in myotubes from controls. The traces in Figure 4B and bar graph plots in Figure 4C show that myotubes from the RMD patients have a significantly smaller (twofold) Ca^2+^ influx peak compared to that obtained in control myotubes.

**Figure 5:**
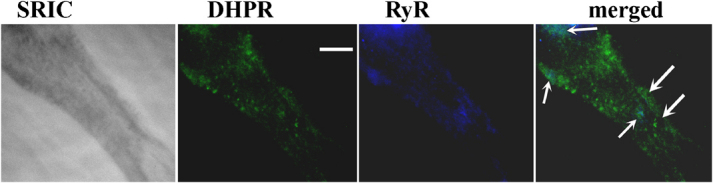
Coimmunolocalization of the α1.1 subunit DHPR and RyR1 by immunofluorescence analysis by TIRF microscopy in human myotubes from a control individual. Myotubes were visualized using an inverted Nikon TE2000 TIRF microscope equipped with a CFI Plan Apochromat 100 × TIRF objective (1.49 NA). Left panel shows photomicrograph of cells through a SRIC filter; central left panel shows the same cells excited with a Sapphire laser at 488 nm (α1.1 subunit of the DHPR; green fluorescence); central right panel shows photomicrograph of the same cells excited at 405 and visualized through a BrightLine CH 427 filter (RyR; dark blue fluorescence). Right panel, merged images using the “color-combine” option included in the Metamorph software package. Arrows indicate overlapping pixels (light blue). Bar indicates 10 µm.

Because the lack of CAV3 is not accompanied by gross alterations in the expression levels of the RyR and DHPRs, the above results indicate that the lack of this protein may affect the topographical distribution of these two Ca^2+^ channels on their respective membranes. To to verify this, we performed immunofluorescence analysis of the distribution of the DHPR and RyR in TIRF mode. Figure 5 shows a representative photomicrograph of a myotube from a control individual observed with a SRIC filter to show that the selected focal plane is at the glass coverslip/membrane interface (left). This focal plane was fixed through the perfect focus system and imunofluorescence analysis was subsequently performed. The central left and right panels of Figure 5 show the punctuated fluorescent distribution of the DHPRα1.1 and RyR on or within 100 nm of the plasma membrane, and the panel on the right shows the merged images revealing areas of colocalization (arrows). Table 1 shows the results of detailed colocalization analysis (*n* = 10 cells): the lack of CAV3 caused a 30% reduction in the area of overlap between the RyR and the DHPR; this was due to a relative increase in the distribution of RyRs in areas not containing DHPRs.

**Table 1:** Colocalization by TIRF Microscopy of the DHPR and RyR in Myotubes from Controls and CAV3^−^ Individuals

Cell phenotype	% Area RyR over DHPR	% Area RyR not over DHPR	% Area DHPR over RyR	% Area DHPR not over RyR
Control	52.6 ± 4.2	47.4 ± 4.2	30.4 ± 3.9	69.7 ± 3.9
RMD (caveolin-3^−^)	36.9 ± 4.8^a^	63.1 ± 4.8^a^	28.0 ± 6.2	71.9 ± 6.8

Colocalization was performed as described in the Materials and Methods section using the “colocalization” option included in the Metamorph 5.7.4 software package.

a *P*<0.03.

## Discussion

In the present study we investigated whether severe reduction of CAV3 as seen in two patients with RMD affects E-C coupling, the physiological process whereby an electrical signal, the depolarization of the muscle fibre, is converted into a chemical signal, that is, release of Ca^2+^ from the sarcoplasmic reticulum, leading to muscle contraction and force development [Fleischer and Inui, 1989; Rios and Pizarro, 1991]. This process depends on the fine micro architecture underlying the calcium release unit whereby the voltage sensing DHPR present on T-tubules faces ordered arrays of RyR1 on the SR junctional membrane [Franzini-Armstrong and Jorgensen, 1994]. Mutations in genes encoding several proteins involved in E-C coupling and Ca^2+^ homeostasis have been shown to be linked to neuromuscular disorders such as Central core disease, multi-minicore disease, Centronuclear myopathy, King Denborough syndrome, and Malignant Hyperthermia [Treves et al., 2005; Wilmshurst et al., 2010; Zhou et al., 2007]. We obtained myotubes from one patient with a homozygous splice site mutation leading to very low levels of caveolin-3 wild-type transcript [Müller et al., 2006]. The other patient harbored the heterozygous p.D28E substitution and also expressed very low levels of caveolin-3; although substitution of an aspartic acid residue for a glutamic acid residue may seem of minor consequence because the two amino acids are negatively charged, mutagenesis studies on other proteins have indicated that such substitutions can lead to protein instability by causing structural perturbations [Mizrahi et al., 1994]. Thus, although the patients harbored different substitutions and had different clinical symptoms, both exhibited a severe reduction in the amount of CAV3 expressed. Thus, by pooling the functional data obtained on myotubes from the two patients, we studied the effect of CAV3 depletion, irrespective of the compensatory mechanisms activated by the patient. The results on cytoplasmic Ca^2+^ homeostasis reveal that the absence of CAV3 does not cause significant alterations of E-C coupling in myotubes and are in agreement with those obtained by Weiss et al. [2008], who showed that loss of caveolin does not affect either the resting [Ca^2+^] or depolarization-induced peak Ca^2+^ release in mouse skeletal muscle cells. A deeper investigation into the electromechanical coupling of caveolin-3-depleted cells, however, revealed a 5-mV shift in the V_1/2_ activating potential, resulting in reduced Ca^2+^ release at low depolarizing potentials. Although different from what was reported in mouse skeletal muscle [Weiss et al., 2008], these results support the findings of Calaghan and White [2006] on rat ventricular myocytes treated with methyl-β-cyclodextrin to disrupt caveolae. In the latter cell type removal of CAV3 resulted in a reduced SR fractional Ca^2+^ release, indicating a loss in E-C coupling efficiency. Thus, as recently suggested by Dart [2010], lipid microdomains may be involved in the fine regulation of ion channels and alterations in the properties and composition of the lipids or alterations in the distribution of caveolins may affect channel gating kinetics, trafficking, and surface expression of proteins [Edelman and Lisanti, 2001].

We were interested in investigating whether the reduced E-C coupling efficiency in *CAV3* mutated cells might have downstream effects. In fact, in a recent study Murata et al. [2007] showed that caveolin-1 is essential for Ca^2+^ entry in endothelial cells; upon stimulation with acetylcholine endothelial cells from caveolin-1 KO mice have a “normal” peak Ca^2+^ transient but a 50% reduction in agonist invoked Ca^2+^ entry. In skeletal muscle cells, E-C coupling is thought to be essentially independent of extracellular Ca^2+^ [Armstrong et al., 1972]. However, a number of recent studies have revealed that activation of the DHPR Ca^2+^ channel is accompanied by influx of Ca^2+^ [Bannister et al., 2009; Cherednichenko et al., 2004], especially during long depolarization. This phenomenon requires the physical presence of the DHPR and RyR1 and is enhanced in cells bearing RyR1 mutations linked to Malignant Hyperthermia [Cherednichenko et al., 2009; Yang et al., 2007]. We found that muscle cells from RMD patients had a 50% reduction in KCl induced Ca^2+^ influx. Western blot analysis revealed no significant differences in the level of expression of DHPR or RyR1; thus, the reduced ECCE is apparently not due to lack of the proteins responsible for the Ca^2+^ influx. One possibility that would explain how the lack of CAV3 decreases ECCE is that lack of CAV3 affects the distribution of the DHPR and RyR1 on their respective membranes. A hypothesis supported by the colocalization experiments performed in TIRF mode as well as by recent results by Vassilopoulos et al. [2010], who showed that RyR1 and caveolin-3 coimmunoprecipitate and that caveolin-3 interacts directly with a transmembrane domain of the RyR1. Thus, the lack of CAV3 seems to derange the microarchitecture of the main protein components of the E-C coupling machinery leading (1) to a less efficient coupling, particularly evident at low depolarizing stimuli and (2) to a decrease in ECCE. We are aware that the TIRF experiments were performed on myotubes and theoretically the colocalization results could reflect a different degree of differentiation of cells from control and RMD patients. To minimalize this possibility, we only analyzed those myotubes containing more than nuclei. In support of our finding, it was demonstrated that skeletal muscles from CAV3 KO mice show abnormalities in the organization of the T-tubules with dilated and longitudinally oriented T-tubules [Galbiati et al., 2001].

Although the results of the present investigation do not explain how the rippling phenomena are induced by passive stretching and percussion, the finding of reduced E-C coupling efficiency and reduced Ca^2+^ influx may explain, at least in part, the phenotypic characteristics of patients with reduced CAV3 levels. Interestingly, Lamb [2005] suggested that the induction of rippling movements may be caused by stretch induced silent action potentials occurring within the T-tubules of skeletal muscle fibers. Although possible, it is experimentally very difficult to prove whether action potentials can escape from T-tubules because cultured myotubes do not differentiate sufficiently in vitro.

In conclusion, we show that loss of caveolin-3 leads to a decrease in the E-C coupling efficiency in human myotubes, and this feature may be one of the underlying causes of the rippling phenotype seen in patients harboring *CAV3* mutations.
