# Supplementary material for: Alterations of Excitation–Contraction Coupling and Excitation Coupled Ca2+ Entry in Human Myotubes Carrying CAV3 Mutations Linked to Rippling Muscle Disease
Source: Hum Mutat. 2011 Feb 3;32(3):309–17. doi: 10.1002/humu.21431 (PMC3132216; doi:10.1002/humu.21431)
Supplement: Supplementary file 1 [file humu0032-0309-SD1.pdf]

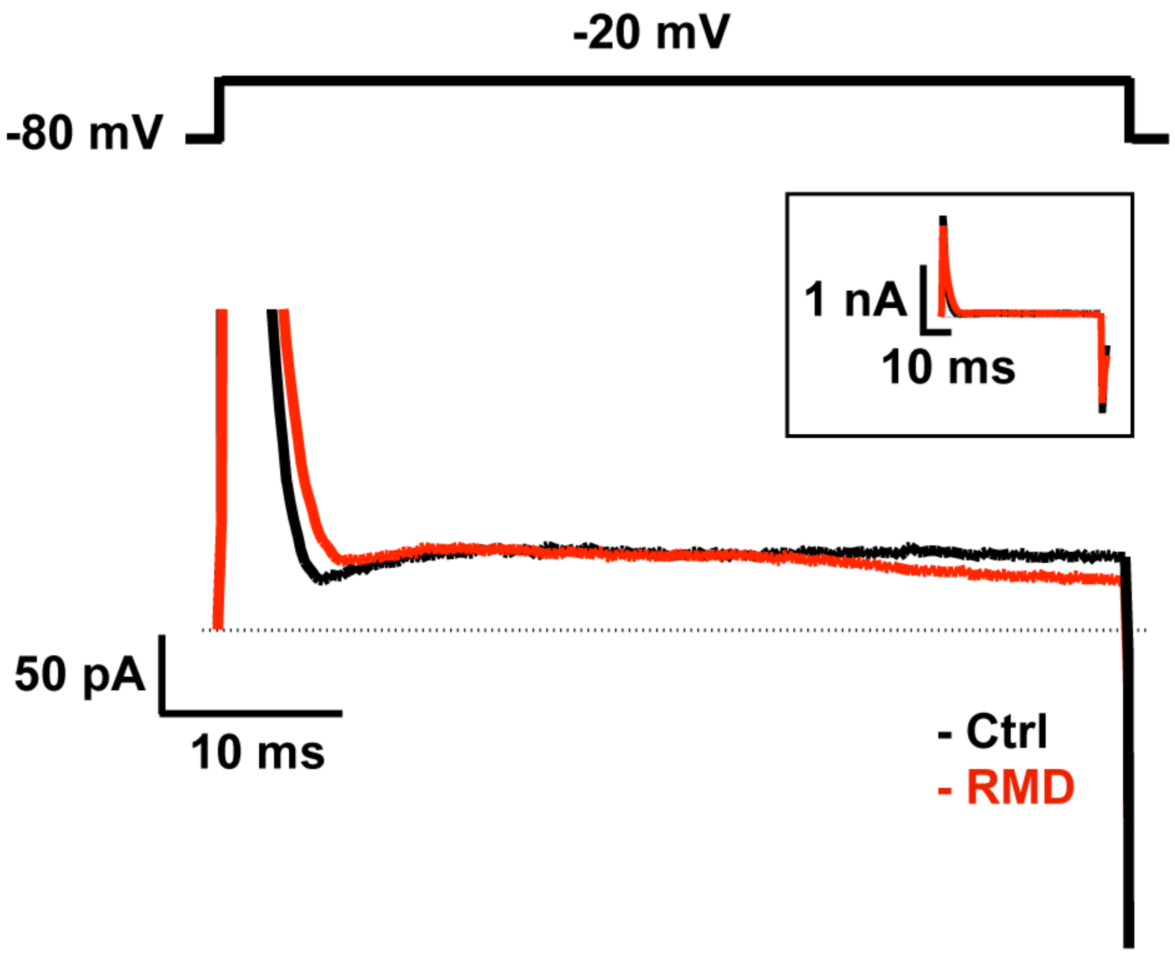

Supp Figure S1. Comparison of voltage-dependent Ca^{2+} currents in control and RMD myotubes. The voltage protocol (upper trace) consists of a 50-ms step from the holding potential (-80 mV) to the test potential (-20 mV). The lower traces show enlarged representative current responses from control (Ctrl, black) and RMD (red) myotubes during depolarization, the full traces are shown in the inset. The dotted line indicates the zero current line. During the 50 ms depolarization, the skeletal Ca^{2+} current may not be maximally activated, but this duration is long enough to activate the voltage sensor of the DHPR and trigger RyR1 activation with subsequent SR Ca^{2+} release. Please note, the current traces show no significant difference in shape or amplitude, however, at this level of depolarization (-20 mV), Ca^{2+} release is significantly reduced in RMD myotubes (see Figure 3 and main text).
